# Bilingual Text Messaging Translation: Translating Text Messages From English Into Spanish for the Text4Walking Program

**DOI:** 10.2196/resprot.3984

**Published:** 2015-05-06

**Authors:** Susan Weber Buchholz, Giselle Sandi, Diana Ingram, Mary Jane Welch, Edith V Ocampo

**Affiliations:** ^1^Rush UniversityCollege of NursingChicago, ILUnited States; ^2^Rush UniversityAcademic AffairsChicago, ILUnited States; ^3^Rush UniversityResearch AffairsChicago, ILUnited States

**Keywords:** text messaging, mobile phone, translating, language, focus groups, exercise

## Abstract

**Background:**

Hispanic adults in the United States are at particular risk for diabetes and inadequate blood pressure control. Physical activity improves these health problems; however Hispanic adults also have a low rate of recommended aerobic physical activity. To address improving physical inactivity, one area of rapidly growing technology that can be utilized is text messaging (short message service, SMS). A physical activity research team, Text4Walking, had previously developed an initial database of motivational physical activity text messages in English that could be used for physical activity text messaging interventions. However, the team needed to translate these existing English physical activity text messages into Spanish in order to have culturally meaningful and useful text messages for those adults within the Hispanic population who would prefer to receive text messages in Spanish.

**Objective:**

The aim of this study was to translate a database of English motivational physical activity messages into Spanish and review these text messages with a group of Spanish speaking adults to inform the use of these text messages in an intervention study.

**Methods:**

The consent form and study documents, including the existing English physical activity text messages, were translated from English into Spanish, and received translation certification as well as Institutional Review Board approval. The translated text messages were placed into PowerPoint, accompanied by a set of culturally appropriate photos depicting barriers to walking, as well as walking scenarios. At the focus group, eligibility criteria for this study included being an adult between 30 to 65 years old who spoke Spanish as their primary language. After a general group introduction, participants were placed into smaller groups of two or three. Each small group was asked to review a segment of the translated text messages for accuracy and meaningfulness. After the break out, the group was brought back together to review the text messages.

**Results:**

A translation confirmation group met at a church site in an urban community with a large population of Hispanics. Spanish speaking adults (N=8), with a mean age of 40 (SD 6.3), participated in the study. Participants were engaged in the group and viewed the text messages as culturally appropriate. They also thought that text messages could motivate them to walk more. Twenty-two new text messages were added to the original database of 246 translated text messages. While the text messages were generally understood, specific word preferences were seen related to personal preference, dialect, and level of formality which resulted in minor revisions to four text messages.

**Conclusions:**

The English text messages were successfully translated into Spanish by a bilingual research staff and reviewed by Hispanic participants in order to inform the use of these text messages for future intervention studies. These Spanish text messages were recently used in a Text4Walking intervention study.

## Introduction

In the United States, Hispanic adults are at increased risk for diabetes and inadequate blood pressure control as compared to white, non-Hispanic adults [1]. Obtaining regular physical activity improves diabetes and hypertension [2]. However, the Hispanic adult population has lower rates of aerobic physical activity (29.1%) compared with the non-Hispanic white population (43.1%) [3]. One area of rapidly growing technology that is being utilized to change health behaviors is SMS text messaging (short message service, SMS) [4]. Intervention studies have shown that text messages can be effectively used in improving physical activity [5,6]. Using text messaging as an intervention to promote physical activity is important to consider in the United States, as 90% of adults use mobile phones and 81% of Americans overall engage in texting. Text messaging is even higher for the Hispanic population (87%) [7], a growing population that has increased by 50% since 2000 in the United States and now represents 53 million Americans [8].

A research team, called Text4Walking has completed formative work in the development of motivational physical activity text messages to be used in physical activity intervention studies. In order to develop an initial database of physical activity text messages in English that could be used for intervention studies, the Text4Walking research team held three focus groups with adults (N=23). To promote group discussion, pictures were used that depicted walking barriers and scenarios. Participants were asked to develop text messages to encourage people to overcome barriers to walking and become more physically active [9]. Additional text messages were later added to this original database by the Text4Walking team.

The research team wanted to include the Hispanic population in their physical activity intervention work because of the low physical activity rates in Hispanics residing in the United States. The vast majority of Hispanic adults (95%) consider it important for future US Hispanic generations to be able to speak Spanish [10]. In addition, Spanish was shown to be the preferred contact language in a longitudinal research program conducted with Mexican Americans to improve diabetes self-management [11]. Therefore, in order for this population to be part of future Text4Walking intervention studies, the team needed to translate existing English text messages into Spanish, to provide participants with a choice of receiving either Spanish or English text messages. However, no study has been located that specifically addresses the process of translating motivational physical activity text messages from English into Spanish.

Translation is an activity that inevitably involves at least two languages and two cultural traditions. The cultural implications for translation may take several forms ranging from lexical content and syntax to ideologies and ways of life in a given culture [12]. Therefore, the translator/facilitator in a research group has to decide on the importance given to certain cultural aspects. Important components to consider with bilingual interventions are bilingual and bicultural facilitators and materials, inclusion of family-based activities, literacy appropriate materials, social support, and a clear understanding of Hispanic cultural values [13].

Federal regulations in the United States require that information about participation in research be presented in a language understandable to the potential subject or their representative [14-16]. The informed consent process is one of the most basic concepts of human subject research. In the *Belmont Report*, the ethical principle of Respect for Persons requires that all subjects be given the opportunity to choose what they will or will not participate in [17]. Consenting requires adequate information, comprehension, and voluntariness. Thus, to meet the requirements of informed consent, if a study’s focus is a population whose principle language is not English, consent documents must be translated into that language. During the consent process an interpreter should be available as well. Each organization’s Institutional Review Board will require verification that the translated consent documents are true translations. Most organizations require a certified translation. It is important for researchers to know how their local policies meet federal requirements [18]. The purpose of this study was to translate a database of English physical activity text messages into Spanish and review those text messages with a group of Spanish speaking adults to inform the use of these text messages in an intervention study.

## Methods

### Design, Sample, and Setting

A translation confirmation group was used for this study [19]. Eligibility criteria for this study included being an adult who spoke Spanish as their primary language, 30 to 65 years old, not engaging in regular physical activity, with no health problems that prohibited them from increasing physical activity, and familiar with texting. The group met at a church site in an urban city with a large population of Hispanics as more than one-fourth (28%) of the city self-identifies as Hispanic [20].

### Procedures

The consent form and study documents, including the existing English physical activity text messages, were translated from English into Spanish initially by a native English speaker fluent in Spanish. These translated messages were then reviewed by a native Spanish speaker fluent in English. These bilingual research team members then gained consensus on the translated documents. The team members used Columbian Spanish for translation. After this, all study documents were reviewed, revised as needed, and approved by a certified translator. Rush University Institutional Review Board approved the study.

The 1.5 hour session was co-moderated by a bilingual doctoral level researcher and a master’s prepared researcher. An English speaking doctoral level experienced focus group researcher was also present. A research assistant recorded participant contributions on a flip chart. The translated text messages were placed into PowerPoint, accompanied by a set of 44 culturally appropriate photos depicting barriers to walking, as well as walking scenarios. Prior to group activity, participants completed a brief survey regarding questions about their text message usage. A general introduction was then provided after which participants were placed into smaller groups of two or three. Each small group was asked to review a segment of the 246 translated text messages for accuracy and meaningfulness. Participants were given handouts with specific translated text messages upon which they were asked to write their comments. After the break out, the group was brought back together to review the text messages.

### Data Analysis

The bilingual group leaders along with an experienced qualitative researcher reviewed three sources of data. First, they reviewed the handwritten participant notes on the handouts. Second, they reviewed the audiotape transcripts that were first transcribed into Spanish and then translated into English. Third, they reviewed the flip chart notes containing group reflections. A consensus was reached by the three researchers who reviewed the data as to when and how to edit any of the translated text messages, as well as determining which text messages should be added as a result of participant suggestions.

## Results

Of the 13 adults screened for the study, 5 were either unable to attend the group or were ineligible. As a result, 8 Spanish speaking adults participated in the study (Table 1).

**Table 1:** Demographics and text message use.

Demographics		
Age (years), mean (SD)		40 (6.3)
Gender (%) – women		63
Ethnicity (%) – Hispanic		100
**Education**		
	Some high school or less (%)	37.5
	Completed high school (%)	37.5
	Some college or completed college (%)	25
Body mass index, mean (SD)		32.25 (5.78)
**Text message use**		
	Mobile phone has text messaging capability (%)	100
	Unlimited text messaging plan (%)	88
	Sends > 4 SMS text messaging weekly (%)	75
	Receives > 4 SMS text messaging weekly (%)	63
**Ease of use of text messaging function (%)**		
	Very easy	50
	Somewhat easy	38
	Neither easy nor difficult	12
	Somewhat difficult	0
	Very difficult	0

Participants were engaged in the group. They thought that text messages could motivate them to walk more and suggested that receiving two text messages a day would be motivational for them. Twenty-two new text messages were added to the original database of 246 translated text messages, which resulted in a total of 268 text messages. While text messages were generally understood and seen as culturally appropriate, specific word preferences were seen related to personal preference, dialect, and level of formality which resulted in minor revisions to four text messages. Table 2 provides examples of 25 of the translated text messages from the approved database.

**Table 2:** Examples of Spanish text messages translated from English.

English text message	Spanish text message
Get up. Today is a good day to walk.	Levántase. Hoy es un buen día para caminar.
Encourage family walking	Anime a la familia a caminar juntos
Activity begins with childhood and never ends	La actividad empieza con la niñez y nunca termina
Get up and start walking	Levántese y empiece a caminar
Walk for peace of mind	Camine para despejar la mente
Enjoy nature – walk	Disfrute de la naturaleza. Camine
Walking is exercise – you can do this!	Caminar es ejercicio - ¡usted puede!
Increase steps today – hike at a park	Aumente sus pasos hoy - tome una caminata en el parque
Get out to walk	Salga a caminar
Eat less. Walk more	Coma menos. Camine más.
Walk with the family	Camine con la familia
Get out and enjoy the day	Salga y disfrute del día
Take some me time – walk	Dedique tiempo para usted
Make leisure time a healthy time	Haga su tiempo libre un tiempo saludable
Schedule time to walk	Reserve tiempo para caminar
Walk around, look around and be safe	Camine, observe y manténgase seguro
Walk and think about life	Camine y piense sobre la vida
Challenge yourself and walk a little further	Póngase la meta de caminar un poco más lejos
Walking daily helps to maintain walking	Caminar diariamente ayuda a mantener el hábito de la caminata
Encourage others to walk with you by exploring as you walk	Anime a otros a que caminen con usted mediante explorar cuando camina
Walk with the kids	Camine con los niños
Relax by walking	Relájese caminando
Take a walk and clear your mind	Salga a caminar y despeje la mente
Get out and move about	Salga y manténgase active
Don't sit still, time doesn't	No se siente por mucho tiempo - el tiempo no espera

## Discussion

This study demonstrated a method whereby English motivational physical activity text messages could be successfully translated into Spanish by a bilingual research team and then reviewed with Hispanic participants in order to inform the use of these text messages in a future intervention study. It is important to use culturally appropriate text messages translated into Spanish to promote healthy behavior changes in the Hispanic population. While intervention sustainability is still a challenge, there is now an opportunity for text messaging programs to be used in the Hispanic population to improve health [21-23]. When ready to be used in the public policy arena, text messages need to be reviewed for both cultural and linguistic appropriateness [24].

This study had some limitations. The sample size was small, from one geographic location, and participants self-selected to be in the study. However, qualitative research is not conducted so that findings can be generalized to other populations. The purpose of this study was to review a translated set of text messages for use in a future intervention study.

Developing culturally appropriate text messages necessitates the use of bilingual and bicultural facilitators and materials to facilitate the development of tailored text messaging [13]. By assuring cultural appropriateness, this study demonstrated an effective method to translate and review physical activity text messages. The research team recently successfully included these Spanish text messages in a Text4Walking intervention study.
